# Association of family history and survival in patients with colorectal cancer: a pooled analysis of eight epidemiologic studies

**DOI:** 10.1002/cam4.1470

**Published:** 2018-03-26

**Authors:** Dawn Q. Chong, Barbara L. Banbury, Amanda I. Phipps, Xinwei Hua, Jonathan Kocarnik, Ulrike Peters, Sonja I. Berndt, Wen‐Yi Huang, John D. Potter, Martha L. Slattery, Emily White, Peter T. Campbell, Tabitha Harrison, Polly A. Newcomb, Andrew T. Chan

**Affiliations:** ^1^ Division of Medical Oncology National Cancer Centre Singapore Singapore Singapore; ^2^ Public Health Sciences Division Fred Hutchinson Cancer Research Center Seattle Washington; ^3^ Department of Epidemiology University of Washington School of Public Health Seattle Washington; ^4^ Institute of Translational Health Sciences University of Washington Seattle Washington; ^5^ Division of Cancer Epidemiology and Genetics National Cancer Institute National Institutes of Health Bethesda Maryland; ^6^ Division of Cancer Epidemiology and Genetics Occupational and Environmental Epidemiology Branch National Cancer Institute National Institutes of Health Bethesda Maryland; ^7^ Centre for Public Health Research Massey University Wellington New Zealand; ^8^ Department of Internal Medicine University of Utah Health Sciences Center Salt Lake City Utah; ^9^ Epidemiology Research Program American Cancer Society Atlanta Georgia; ^10^ Division of Gastroenterology Massachusetts General Hospital and Harvard Medical School Boston Massachusetts; ^11^ Clinical and Translational Epidemiology Unit Massachusetts General Hospital and Harvard Medical School Boston Massachusetts; ^12^ Department of Medicine Channing Division of Network Medicine Brigham and Women's Hospital and Harvard Medical School Boston Massachusetts

**Keywords:** Colorectal cancer, family history, first‐degree relative, mortality, survival

## Abstract

A family history of colorectal cancer (CRC) in first‐degree relatives (FDRs) increases the risk of CRC. However, the influence of family history on survival among CRC patients remains unclear. We conducted a pooled analysis of survival in 5010 incident CRC cases. Cox proportional hazards models were used to estimate the association of family history with overall survival (OS) and CRC‐specific survival (CSS). We also assessed the impact of the number of affected FDRs and age at CRC diagnosis in the affected FDRs on survival. Among CRC cases, 819 (16%) patients reported a family history of CRC. There were 1580 total deaths over a median follow‐up of 4.6 years, of which 1046 (66%) deaths were due to CRC. Having a family history of CRC was not associated with OS [hazard ratio (HR), 1.03; 95% confidence interval (CI), 0.89–1.19] or CSS (HR, 1.13; 95% CI, 0.95–1.36)]. There were no associations between the number of affected relatives or age at CRC diagnosis of the affected relative with survival (all *P*_trend_ > 0.05). However, a family history of CRC did confer worse CSS in patients diagnosed with distal colon cancer (HR, 1.45, 95% CI, 1.03–2.04). A family history of CRC was generally not associated with survival after CRC diagnosis. However, having a family history of CRC was associated with worse CRC prognosis in individuals with distal colon cancer, suggesting a possible genetic predisposition with distinct pathogenic mechanism that may lead to worse survival in this group.

## Introduction

Colorectal cancer (CRC) is the third most common cancer diagnosed in both men and women in the United States. CRC is the second leading cause of cancer‐related deaths in men and the third leading cause in women in the United States 1. It is characterized by high heritability with approximately 10–15% of CRC patients having an affected first‐degree relative (FDR), defined as the presence of CRC in a parent, sibling, or child 2, 3, 4. Individuals with a family history of CRC in FDRs have a twofold to threefold increased risk of developing CRC 2, 5, 6. This risk increases with a greater number of affected FDRs, the closer the degree of relation and younger age at diagnosis 2, 5, 6, 7, 8.

Highly penetrant hereditary CRC syndromes, including familial adenomatous polyposis (FAP) and Lynch syndrome account for less than 5% of the familial risk 9, 10. Beyond these rare CRC syndromes that have been associated with favorable prognosis 11, 12, the effect of general family history on CRC survival remains inconsistent 6, 13, 14, 15, 16, 17, 18, 19, 20, 21, 22, 23, 24, 25. Several studies reported no association between having a family history of CRC and survival 16, 18, 24, 25. Among 4284 CRC cases within the Colon Cancer Family Registry (CCFR), Phipps et al. 18 reported no association between family history and overall survival (OS) [hazard ratio (HR), 0.92; 95% confidence interval (CI), 0.79–1.08] or disease‐specific survival (HR, 1.03; 95% CI, 0.85–1.24). Another study conducted among 2236 individuals within the Utah Population Database also revealed no impact of family history on survival 24. In contrast, other studies reported either favorable or worse prognosis among patients with a family history of CRC 14, 15, 17, 19, 22. In a large retrospective study of 10,782 CRC cases, Morris et al. 17 demonstrated that patients with a family history of CRC had a 11% lower risk of death compared to those with sporadic CRC (HR, 0.89; 95% CI, 0.81–0.98). Conversely, an analysis of 2090 incident CRC cases within the Prostate, Lung, Colorectal, and Ovarian Cancer Screening Trial (PLCO) showed an increased risk of CRC‐specific mortality (HR, 1.31; 95% CI, 1.02–1.69) in individuals with a family history of CRC, compared to those without a family history 19. The inconsistent evidence may be attributed to varied study designs (population‐based cohort studies compared to case–control studies) or baseline differences in characteristics of the study population. Hence, we aim to address these inconsistencies by examining the influence of family history of CRC on OS and CRC‐specific survival (CSS) after CRC diagnosis using individual patient data in eight observational epidemiologic studies in the International Survival Analysis in Colorectal Cancer Consortium (ISACC).

## Methodology

### Study population

We conducted a pooled analysis of 5010 incident CRC cases from six prospective cohort studies (Cancer Prevention Study‐II [CPS‐II] 26, Health Professionals Follow‐up Study [HPFS] 27, Nurses’ Health Study [NHS] 28, Prostate, Lung, Colorectal, and Ovarian Cancer Screening Trial [PLCO] 29, VITamins And Lifestyle study [VITAL] 30, Women's Health Initiative [WHI] 31), and two population‐based case–control studies (Post‐Menopausal Hormone Study within the Colorectal Cancer Family Registry [PMH‐CCFR] 32, Diet, Activity, and Lifestyle Study [DALS] 33) within ISACC. We included patients with incident CRC who provided information regarding family history of CRC in FDRs. We excluded individuals with a history of cancers other than nonmelanoma skin cancers. This study was approved by the Institutional Review Boards of the respective institutions. Informed consent was obtained from all participants.

### Data collection

Demographic and epidemiologic data were obtained either at enrollment into the respective study (PMH‐CCFR, CPS‐II, DALS, PLCO, VITAL, WHI) or at subsequent follow‐up at time of blood draw (HPFS, NHS). Family history of CRC, defined as either a parent, sibling, or child diagnosed with CRC was also collected as part of study questionnaire. All studies collected information on the number of FDRs with CRC. Several studies (PMH‐CCFR, CPS‐II, DALS, and PLCO) collected information regarding the age at diagnosis of CRC in the affected FDRs. Incident CRC was identified through self‐report with medical record review (CPS‐II, PLCO), self‐report with medical and pathological records review (NHS, HPFS), central physician adjudication (WHI), or linkage to the Surveillance, Epidemiology, and End Results (SEER) cancer registry (PMH‐CCFR, CPS‐II, DALS, VITAL). Vital status was ascertained in all studies through follow‐up and linkages to the National Death Index (NDI), except for VITAL, which was ascertained via linkage with the Washington State death records with censoring when a participant moved out of Washington State.

### Statistical analyses

Missing data for smoking status, body mass index (BMI), regular use of aspirin or nonsteroidal anti‐inflammatory drugs (NSAIDs), history of screening endoscopy and stage at diagnosis (all less than 6%) was accounted for by mean and mode imputation. Descriptive statistics were generated by standard methods. OS was defined as the time from diagnosis of CRC to the time of death from any cause. CSS was defined as the time from diagnosis of CRC to the time of death due to CRC. Cox proportional hazards (PH) models were used to estimate the HR and 95% CI for the association between family history of CRC with OS and CSS. PH assumption for the main effects was verified using Schoenfeld residuals. We stratified on the variables that violated the PH assumption, which include tumor location, stage at diagnosis (based on AJCC staging), and study site.

We fitted separate models for the associations of family history of CRC in FDR (yes/no), number of FDR with a history of CRC (0/1/≥2), youngest age at diagnosis of affected FDR (<60 years, ≥60 years), with OS and CSS. All analyses were adjusted for age at diagnosis (<60 years, 60–<70 years, ≥70 years), sex, BMI (<25 kg/m^2^, 25‐<30 kg/m^2^, ≥30 kg/m^2^), smoking status (never, former, current), regular use of aspirin or other NSAIDs 34, and history of screening endoscopy at baseline (yes/no), and stratified by study site. Additional analyses were adjusted for the covariates above, and stratified by stage at diagnosis (I, II/III, IV), and tumor location (proximal colon, distal colon, rectum, other), and study site. We performed prespecified subgroup analyses by age at diagnosis (<60 years, ≥60 years), sex, stage at diagnosis (I/II/III, IV), and tumor location (proximal colon, distal colon, rectum). Tests of interactions between family history of CRC and potentially modifying covariates were assessed by entering the cross‐product term for FDR and the covariate of interest in the multivariate models. Two‐tailed *P*‐value of <0.05 was considered statistically significant. All analyses were performed using R version 3.2.2 and SAS version 9.4 (SAS Institute, Inc, Cary, NC).

## Results

Of the 5010 eligible participants, 819 (16.3%) individuals reported a family history of CRC in one or more FDRs at the baseline interview. The median follow‐up period was 4.6 years (interquartile range 2.2–7.3 years) after CRC diagnosis. Among the 1580 (31.5%) total deaths, 1046 (66.2%) deaths were attributed to CRC. Individuals who reported a first‐degree family history of CRC were more likely to have undergone screening endoscopy (*P* < 0.001) and have proximal colon tumors (*P* = 0.04) and nonmetastatic CRC (*P* = 0.003; Table 1). Overall, having a family history of CRC was not associated with OS (HR, 1.03; 95% CI, 0.89–1.19) or CSS (HR, 1.13; 95% CI, 0.95–1.36) after adjusting for age at diagnosis, sex, BMI, smoking status, regular use of aspirin or NSAIDs, history of endoscopy screening, and stratified by tumor location, stage at diagnosis, and study site (Table 2).

**Table 1:** Baseline characteristics of study participants according to family history of colorectal cancer

Characteristic	No family history of CRC (*n* = 4191) *N* (%)	Positive family history of CRC (*n* = 819) *N* (%)
Age at diagnosis, year		
<40	22 (1)	6 (1)
40–<50	67 (2)	23 (3)
50–<60	461 (11)	80 (10)
60–<70	1612 (39)	286 (35)
≥70	2029 (48)	424 (52)
Median age at diagnosis, year (Interquartile range)	71 (65,76)	70 (65,75)
Sex		
Male	1534 (37)	281 (34)
Female	2657 (63)	538 (66)
Smoking status		
Never	1842 (44)	344 (42)
Former	1928 (46)	399 (49)
Current	398 (10)	71 (9)
Unknown	23 (1)	5 (1)
Body mass index, kg/m^2^		
<25.0	1441 (34)	283 (35)
25.0–29.9	1629 (39)	324 (40)
≥30.0	1067 (26)	205 (25)
Unknown	54 (1)	7 (1)
Regular Aspirin/NSAIDS use		
Yes	1496 (36)	294 (36)
No	2571 (61)	508 (62)
Unknown	124 (3)	17 (2)
History of prior screening endoscopy		
Yes	1514 (36)	359 (44)
No	2430 (58)	418 (51)
Unknown	247 (6)	42 (5)
Tumor site		
Proximal colon	2107 (50)	437 (53)
Distal colon	1360 (33)	240 (29)
Rectum	632 (15)	114 (14)
Others	92 (2)	28 (3)
Stage of CRC		
I	1415 (34)	306 (37)
II/III	2036 (49)	398 (49)
IV	512 (12)	67 (8)
Unknown	228 (5)	48 (6)
Study site		
CPS‐II	529	82
HPFS	131	36
NHS	250	46
PLCO	853	148
VITAL	233	40
WHI	1011	257
CCFR‐PMH	259	20
DALS	925	190

CRC, colorectal cancer; NSAIDs, nonsteroidal anti‐inflammatory drugs.

**Table 2:** Family history of colorectal cancer in first‐degree relatives and overall and colorectal cancer‐specific survival after colorectal cancer diagnosis

	Overall survival			Colorectal cancer‐specific survival		
	No. of deaths/no. at risk	Model 1 HR (95% CI)^a^	Model 2 HR (95% CI)^b^	No. of deaths/no. at risk	Model 1 HR (95% CI)^a^	Model 2 HR (95% CI)^b^
Family history in FDR						
No	1337/4191	1.0 (ref)	1.0 (ref)	885/4190	1.0 (ref)	1.0 (ref)
Yes	243/819	0.90 (0.78–1.03)	1.03 (0.89–1.19)	161/819	0.91 (0.77–1.07)	1.13 (0.95–1.36)
No. of affected FDR						
0	1337/4191	1.0 (ref)	1.0 (ref)	885/4191	1.0 (ref)	1.0 (ref)
1	180/627	0.92 (0.78–1.07)	1.00 (0.85–1.18)	122/627	0.95 (0.79–1.15)	1.11 (0.91–1.37)
≥2	21/83	0.73 (0.47–1.13)	0.92 (0.59–1.43)	12/83	0.68 (0.38–1.21)	0.98 (0.55–1.76)
Age at CRC diagnosis of affected FDR^c^						
No family history	754/2566	1.0 (ref)	1.0 (ref)	476/2566	1.0 (ref)	1.0 (ref)
<60 years	28/118	0.73 (0.50–1.07)	0.84 (0.57–1.24)	20/118	0.85 (0.54–1.33)	1.09 (0.69–1.74)
≥60 years	91/289	1.01 (0.82–1.26)	1.02 (0.81–1.29)	58/289	1.08 (0.82–1.42)	1.19 (0.89–1.61)

CRC, colorectal cancer; FDR, first‐degree relative; HR, hazard ratio; CI, confidence interval.

a Adjusted for age at diagnosis, sex, body mass index, smoking, regular use of aspirin or nonsteroidal anti‐inflammatory drugs, history of endoscopy screening and stratified by study site.

b Adjusted for age at diagnosis, sex, body mass index, smoking, regular use of aspirin or nonsteroidal anti‐inflammatory drugs, history of endoscopy screening, and stratified by tumor location, stage at diagnosis, and study site.

c Age at CRC diagnosis of the youngest affected FDR.

There was no evidence of an association between the number of affected FDRs on survival. Compared to patients without a family history of CRC, the multivariate HRs for OS were 1.00 (95% CI, 0.85–1.18) for individuals with 1 affected FDR and 0.92 (95% CI, 0.59–1.43) for individuals with ≥2 affected FDRs. The corresponding HRs for CSS were 1.11 (95% CI, 0.91–1.37) with 1 affected FDR and 0.98 (95% CI, 0.55–1.76) for ≥2 affected FDRs (Table 2). There was also no relationship between the age at CRC diagnosis of the affected FDR with OS or CSS. Compared to patients without a family history of CRC, the HRs for OS were 0.84 (95% CI, 0.57–1.24) for individuals with an affected FDR diagnosed at age <60 years, and 1.02 (95% CI, 0.81–1.29) for those with an affected FDR diagnosed at age ≥60 years. The corresponding HRs for CSS were 1.09 (95% CI, 0.69–1.74) and 1.19 (95% CI, 0.89–1.61), respectively (Table 2).

In subgroup analyses, the effect of family history appeared to vary according to tumor location (Table 3). Among patients with distal colon cancer, having a family history of CRC was associated with lower CSS (HR, 1.45; 95% CI, 1.03–2.04), compared to individuals without a family history of CRC. In contrast, there was no statistically significant association between family history of CRC and CSS among individuals with proximal colon cancer (HR, 1.04; 95% CI, 0.82–1.33) or rectal cancer (HR, 1.34; 95% CI, 0.80–2.23).

**Table 3:** Subgroup analyses of overall and colorectal cancer‐specific survival by family history of colorectal cancer

		Overall survival			Colorectal cancer‐specific survival		
		No family history of CRC	Positive family history of CRC	*P*_int_	No family history of CRC	Positive family history of CRC	*P*_int_
Age at diagnosis							
<60 years	No. of deaths/no. at risk	134/462	19/90	0.13	110/462	17/90	0.46
	MV HR (95% CI)	1.0 (ref)	0.62 (0.33–1.14)		1.0 (ref)	0.64 (0.33–1.25)	
≥60 years	No. of deaths/no. at risk	1203/3729	224/729		775/3729	144/729	
	MV HR (95% CI)	1.0 (ref)	1.07 (0.92–1.24)		1.0 (ref)	1.18 (0.98–1.43)	
Sex							
Male	No. of deaths/no. at risk	519/1534	88/281	0.55	305/1534	57/281	0.38
	MV HR (95% CI)	1.0 (ref)	0.94 (0.74–1.21)		1.0 (ref)	1.25 (0.91–1.71)	
Female	No. of deaths/no. at risk	818/2657	155/538		580/2657	104/538	
	MV HR (95% CI)	1.0 (ref)	1.08 (0.90–1.30)		1.0 (ref)	1.10 (0.88–1.37)	
Stage							
I/II/III	No. of deaths/no. at risk	810/3451	169/704	0.21	416/3451	100/704	0.77
	MV HR (95% CI)	1.0 (ref)	0.97 (0.83–1.14)		1.0 (ref)	1.07 (0.87–1.33)	
IV	No. of deaths/no. at risk	437/512	58/67		412/512	53/67	
	MV HR (95% CI)	1.0 (ref)	1.28 (0.94–1.73)		1.0 (ref)	1.25 (0.91–1.73)	
Tumor site							
Proximal colon	No. of deaths/no. at risk	716/2107	132/437	0.67	482/2107	83/437	0.34
	MV HR (95% CI)	1.0 (ref)	1.01 (0.83–1.22)		1.0 (ref)	1.04 (0.82–1.33)	
Distal colon	No. of deaths/no. at risk	381/1360	67/240		235/1360	47/240	
	MV HR (95% CI)	1.0 (ref)	1.15 (0.87–1.51)		1.0 (ref)	1.45 (1.03–2.04)	
Rectum	No. of deaths/no. at risk	194/632	31/114		138/632	21/114	
	MV HR (95% CI)	1.0 (ref)	1.29 (0.85–1.96)		1.0 (ref)	1.34 (0.80–2.23)	

MV HR, multivariate hazard ratio; CI, confidence interval.

Multivariate model adjusted for age at diagnosis, sex, body mass index, smoking, regular use of aspirin or nonsteroidal anti‐inflammatory drugs, history of endoscopy screening, study site, tumor location, and stage at diagnosis, with the omission of the stratification variable in the model.

## Discussion

In this large pooled analysis of 5010 CRC cases, having a family history of CRC in FDRs was not associated with CRC survival in the overall population. There were also no significant associations between the number of affected FDRs or age at CRC diagnosis of the affected FDRs and CRC survival. Compared to patients without a family history of CRC, having a family history of CRC in FDRs among individuals with distal colon conferred worse CSS.

Having a family history of CRC is a known risk factor for the development of CRC 2, 5, 6, 19. However, the prognostic significance of family history of CRC remains uncertain 13, 14, 15, 16, 17, 18, 19, 20, 22, 23, 24, 25. Similar to previous studies, we found that individuals with a family history of CRC were more likely to have proximal colon cancer 18, earlier stage at diagnosis 6, 13, and underwent screening endoscopy 13, 19. However, this did not lead to differences in CSS and OS in our adjusted analyses. Our finding of no association between family history of CRC and survival in patients with CRC is consistent with reports from five previous studies conducted in population‐based cohorts or within a clinical trial 16, 18, 23, 24, 25. The most robust study was conducted within the CCFR which reported no association between family history of CRC and OS, after adjusting for microsatellite instability (MSI) status or tumor site 18. Although we are unable to account for MSI and other mutation status, for example, V‐Ki‐ras2 Kirsten rat sarcoma viral oncogene homolog (*KRAS*) due to lack of information, several studies suggested that adjustment for MSI or mismatch repair (MMR) status and mutation status had minimal impact on the null associations 18, 25. Among stage III resected colon cancer patients treated with adjuvant chemotherapy in a randomized controlled trial (N0147), having a family history of CRC did not impact disease free survival and OS, after adjustment for other predictors of mortality including *KRAS* or *BRAF* mutations and MMR status 25.

Our results contradict two large studies conducted within the PLCO which reported a 31% increase risk of mortality, and a population‐based registry in the United Kingdom which reported a modest 11% reduction in the risk of death in individuals with a family history of CRC compared to those with sporadic CRC 17, 19. The PLCO study included individuals aged 55–74 years, whereas our study included patients beyond this age range, hence selection bias could account for some of the observed differences. In addition, the PLCO study did not adjust for stage and smoking which are important predictors of mortality 35, 36. Similar to the PLCO study and another study conducted within the NHS, younger age at diagnosis in the affected FDR (<60 years) was not associated with differential increase risk of CRC mortality compared to individuals with FDRs diagnosed at older ages in our study 13, 19. In the retrospective study conducted by Morris et al. 17 within the National Study of CRC Genetics (NSCCG), individuals with familial CRC had improved survival compared to those with sporadic CRC, with survival correlating with the number of affected FDRs. The observed differences may be attributed to different population characteristics and study design. The NSCCG was a retrospective observational study with inherent recall bias, whereas most of the studies included in our study are prospective cohort studies. Compared to our study, the patients within the NSCCG were younger and had a higher proportion of rectal cancer. It is also unknown whether there were differences in other predictors of mortality such as smoking, BMI, prior endoscopy, use of aspirin/NSAIDs between individuals with and without a family history of CRC within the NSCCG, and these confounders were not accounted for in their analyses. Although the authors suggested that the improved survival may be due to a higher proportion of cases with right‐sided tumors among those with a family history of CRC, which are associated with a deficient MMR pathway linked to better prognosis 37, 38, the MMR status in the CRC cases were not available for analyses. In contrast, we did not see improved survival in our cohort despite having a higher proportion of right‐sided tumors in those with family history of CRC, which was consistent with the study conducted within CCFR 18. Furthermore, there remains a conundrum regarding the prognosis of right‐sided tumors as they are also commonly associated with BRAF mutations which confer a poor prognosis 39, 40. Family history of CRC has been shown to confer an improved OS in stage III colon cancer patients with deficient MMR expressing tumors compared to proficient MMR tumors 25. The impact of MSI and mutational status on the prognostic significance of family history certainly warrants further validation.

In our study, having a family history of CRC in FDRs was associated with increased CRC‐specific mortality in individuals with distal colon cancer, but not proximal or rectal cancers. For individuals with rectal cancers, there was a trend toward worse CSS although not significant, possibly due to limited statistical power. Our finding differs from the result of the NHS study which showed an increased risk of CRC mortality among those with proximal colon cancer (HR 1.69; 95% CI, 1.03–2.77) 13. The NHS cohort comprises of females only and this may limit its comparability to our study that included both males and females. The increased mortality observed in patients with distal colon cancer with a family history of CRC in our study may suggest a biologically more aggressive disease due to differences in molecular and genetic features or shared environmental factors among FDRs. These possibilities warrant further investigation.

Our study has several strengths. First, this analysis included a large study population of CRC cases (*n* = 5010) with detailed demographic and epidemiologic information. Second, most of the included studies are prospective cohort studies, which minimized the possibility of recall bias attributed to differential reporting of family history of CRC with varying cancer stages. Third, we had information on the number of affected FDRs and age at diagnosis of CRC in affected FDRs in a subset of the population. This allowed us to explore the influence of the number of affected FDRs and age at CRC diagnosis of the affected FDR on survival.

There are some limitations in this study. First, family history information was obtained by self‐report. This may result in a potential for misclassification of family history status. However, self‐report family history has been shown to be reliable when compared to death certificates, medical records, or registry data 41, 42. Second, we could not exclude cases with familial cancer syndromes (FAP and Lynch syndrome) from all studies as most of the studies did not elicit this information. However, this is unlikely to alter our results significantly as familial CRC syndromes account for less than 5% of CRC cases 10. Third, although we were unable to account for the effect of CpG island methylator phenotype (CIMP), MSI status, and mutations such as *BRAF* due to lack of available information, which are known to affect CRC prognosis, studies have suggested that adjustment of these factors had little impact on the associations 18, 25. Last, the limited availability of treatment data precludes adjustment for treatment in the multivariate models. However, treatment was generally uniform according to stage at diagnosis. Thus, our adjustment for stage at diagnosis should largely minimize any different effect of treatment on survival.

In conclusion, we found that having a family history of CRC in FDRs was generally not associated with OS and CSS, after adjusting for other predictors of mortality including stage at diagnosis. However, our data indicate that family history of CRC may be associated with worse CRC prognosis in patients with distal colon cancer, suggesting a possible genetic predisposition with distinct pathogenic mechanism that confers a poor prognosis. Additional studies are needed to validate our findings and fully elucidate the potential pathways by which family history may affect survival.

## Conflict of Interest

None declared.

## References

[1] American Cancer Society . Cancer Facts & Figures 2017.

[2] Fuchs, C. S. , E. L. Giovannucci , G. A. Colditz , D. J. Hunter , F. E. Speizer , and W. C. Willett . 1994 A prospective study of family history and the risk of colorectal cancer. N. Engl. J. Med. 331:1669–1674. 7969357 10.1056/NEJM199412223312501

[3] Ramsey, S. D. , P. Yoon , R. Moonesinghe , and M. J. Khoury . 2006 Population‐based study of the prevalence of family history of cancer: implications for cancer screening and prevention. Genet. Med. 8:571–575. 16980813 10.1097/01.gim.0000237867.34011.12 PMC2726801

[4] Lynch, H. T. , and A. de la Chapelle . 2003 Hereditary colorectal cancer. N. Engl. J. Med. 348:919–932. 12621137 10.1056/NEJMra012242

[5] Johns, L. E. , and R. S. Houlston . 2001 A systematic review and meta‐analysis of familial colorectal cancer risk. Am. J. Gastroenterol. 96:2992–3003. 11693338 10.1111/j.1572-0241.2001.04677.x

[6] Slattery, M. L. , and R. A. Kerber . 1994 Family history of cancer and colon cancer risk: the Utah Population Database. J. Natl Cancer Inst. 86:1618–1626. 7932826 10.1093/jnci/86.21.1618

[7] Stephenson, B. M. , P. J. Finan , J. Gascoyne , F. Garbett , V. A. Murday , and D. T. Bishop . 1991 Frequency of familial colorectal cancer. Br. J. Surg. 78:1162–1166. 1958974 10.1002/bjs.1800781005

[8] Butterworth, A. S. , J. P. Higgins , and P. Pharoah . 2006 Relative and absolute risk of colorectal cancer for individuals with a family history: a meta‐analysis. Eur. J. Cancer 42:216–227. 16338133 10.1016/j.ejca.2005.09.023

[9] Peel, D. J. , A. Ziogas , E. A. Fox , M. Gildea , B. Laham , E. Clements , et al. 2000 Characterization of hereditary nonpolyposis colorectal cancer families from a population‐based series of cases. J. Natl Cancer Inst. 92:1517–1522. 10995807 10.1093/jnci/92.18.1517

[10] Potter, J. D. 1999 Colorectal cancer: molecules and populations. J. Natl Cancer Inst. 91:916–932. 10359544 10.1093/jnci/91.11.916

[11] Watson, P. , K. M. Lin , M. A. Rodriguez‐Bigas , T. Smyrk , S. Lemon , M. Shashidharan , et al. 1998 Colorectal carcinoma survival among hereditary nonpolyposis colorectal carcinoma family members. Cancer 83:259–266. 9669808

[12] Gryfe, R. , H. Kim , E. T. Hsieh , M. D. Aronson , E. J. Holowaty , S. B. Bull , et al. 2000 Tumor microsatellite instability and clinical outcome in young patients with colorectal cancer. N. Engl. J. Med. 342:69–77. 10631274 10.1056/NEJM200001133420201

[13] Bass, A. J. , J. A. Meyerhardt , J. A. Chan , E. L. Giovannucci , and C. S. Fuchs . 2008 Family history and survival after colorectal cancer diagnosis. Cancer 112:1222–1229. 18219663 10.1002/cncr.23294

[14] Birgisson, H. , A. Ghanipour , K. Smedh , L. Pahlman , and B. Glimelius . 2009 The correlation between a family history of colorectal cancer and survival of patients with colorectal cancer. Fam. Cancer 8:555–561. 19714489 10.1007/s10689-009-9286-0

[15] Chan, J. A. , J. A. Meyerhardt , D. Niedzwiecki , D. Hollis , L. B. Saltz , R. J. Mayer , et al. 2008 Association of family history with cancer recurrence and survival among patients with stage III colon cancer. JAMA 299:2515–2523. 18523220 10.1001/jama.299.21.2515 PMC3616330

[16] Kirchhoff, A. C. , P. A. Newcomb , A. Trentham‐Dietz , H. B. Nichols , and J. M. Hampton . 2008 Family history and colorectal cancer survival in women. Fam. Cancer 7:287–292. 18360806 10.1007/s10689-008-9190-z

[17] Morris, E. J. A. , S. Penegar , L. E. Whitehouse , P. Quirke , P. Finan , D. T. Bishop , et al. 2013 A retrospective observational study of the relationship between family history and survival from colorectal cancer. Br. J. Cancer 108:1502–1507. 23511565 10.1038/bjc.2013.91 PMC3629434

[18] Phipps, A. I. , D. J. Ahnen , P. T. Campbell , A. K. Win , M. A. Jenkins , N. M. Lindor , et al. 2014 Family history of colorectal cancer is not associated with colorectal cancer survival regardless of microsatellite instability status. Cancer Epidemiol. Biomarkers Prev. 23:1700–1704. 24891550 10.1158/1055-9965.EPI-14-0533 PMC4119483

[19] Schoen, R. E. , A. Razzak , J. Y. Kelly , S. I. Berndt , K. Firl , T. L. Riley , et al. 2015 Incidence and mortality of colorectal cancer in individuals with a family history of colorectal cancer. Gastroenterology 149:1438–1445.e1431. 26255045 10.1053/j.gastro.2015.07.055 PMC4628587

[20] Zell, J. A. , J. Honda , A. Ziogas , and H. Anton‐Culver . 2008 Survival after colorectal cancer diagnosis is associated with colorectal cancer family history. Cancer Epidemiol. Biomarkers Prev. 17:3134–3140. 18990755 10.1158/1055-9965.EPI-08-0587 PMC2739671

[21] Kao, P. S. , J. K. Lin , H. S. Wang , S. H. Yang , J. K. Jiang , W. S. Chen , et al. 2009 The impact of family history on the outcome of patients with colorectal cancer in a veterans’ hospital. Int. J. Colorectal Dis. 24:1249–1254. 19621228 10.1007/s00384-009-0774-3

[22] Lee, S. D. , B. C. Kim , K. S. Han , C. W. Hong , D. K. Sohn , J. W. Park , et al. 2014 Influence of family history on survival in patients with colon and rectal cancer. J. Dig. Dis. 15:108–115. 24304621 10.1111/1751-2980.12120

[23] Kune, G. A. , S. Kune , and L. F. Watson . 1992 The effect of family history of cancer, religion, parity and migrant status on survival in colorectal cancer. The Melbourne Colorectal Cancer Study. Eur. J. Cancer 28A:1484–1487. 1515272 10.1016/0959-8049(92)90549-h

[24] Slattery, M. L. , and R. A. Kerber . 1995 The impact of family history of colon cancer on survival after diagnosis with colon cancer. Int. J. Epidemiol. 24:888–896. 8557444 10.1093/ije/24.5.888

[25] Jansson‐Knodell, C. L. , N. R. Foster , D. J. Sargent , P. J. Limburg , S. N. Thibodeau , T. C. Smyrk , et al. 2017 Family history of colorectal cancer and its impact on survival in patients with resected stage III colon cancer: results from NCCTG Trial N0147 (Alliance). J. Gastrointest. Oncol. 8:1–11. 28280603 10.21037/jgo.2016.12.13 PMC5334054

[26] Campbell, P. T. , C. C. Newton , A. N. Dehal , E. J. Jacobs , A. V. Patel , and S. M. Gapstur . 2012 Impact of body mass index on survival after colorectal cancer diagnosis: the Cancer Prevention Study‐II Nutrition Cohort. J. Clin. Oncol. 30:42–52. 22124093 10.1200/JCO.2011.38.0287

[27] Coakley, E. H. , E. B. Rimm , G. Colditz , I. Kawachi , and W. Willett . 1998 Predictors of weight change in men: results from the Health Professionals Follow‐up Study. Int. J. Obes. Relat. Metab. Disord. 22:89–96. 9504316 10.1038/sj.ijo.0800549

[28] Belanger, C. F. , C. H. Hennekens , B. Rosner , and F. E. Speizer . 1978 The nurses’ health study. Am. J. Nurs. 78:1039–1040. 248266

[29] Gohagan, J. K. , P. C. Prorok , R. B. Hayes , B. S. Kramer , L. C. Prostate , and Ovarian Cancer Screening Trial Project T . 2000 The Prostate, Lung, Colorectal and Ovarian (PLCO) Cancer Screening Trial of the National Cancer Institute: history, organization, and status. Control. Clin. Trials 21(6 Suppl.):251S–272S. 11189683 10.1016/s0197-2456(00)00097-0

[30] White, E. , R. E. Patterson , A. R. Kristal , M. Thornquist , I. King , A. L. Shattuck , et al. 2004 VITamins and lifestyle cohort study: study design and characteristics of supplement users. Am. J. Epidemiol. 159:83–93. 14693663 10.1093/aje/kwh010

[31] Hays, J. , J. R. Hunt , F. A. Hubbell , G. L. Anderson , M. Limacher , C. Allen , et al. 2003 The Women's Health Initiative recruitment methods and results. Ann. Epidemiol. 13(9 Suppl.):S18–S77. 14575939 10.1016/s1047-2797(03)00042-5

[32] Newcomb, P. A. , J. Baron , M. Cotterchio , S. Gallinger , J. Grove , R. Haile , et al. 2007 Colon Cancer Family Registry: an international resource for studies of the genetic epidemiology of colon cancer. Cancer Epidemiol. Biomarkers Prev. 16:2331–2343. 17982118 10.1158/1055-9965.EPI-07-0648

[33] Slattery, M. L. , J. Potter , B. Caan , S. Edwards , A. Coates , K. N. Ma , et al. 1997 Energy balance and colon cancer–beyond physical activity. Cancer Res. 57:75–80. 8988044

[34] Nan, H. , C. M. Hutter , Y. Lin , E. J. Jacobs , C. M. Ulrich , E. White , et al. 2015 Association of aspirin and NSAID use with risk of colorectal cancer according to genetic variants. JAMA 313:1133–1142. 25781442 10.1001/jama.2015.1815 PMC4382867

[35] Ordonez‐Me, J. M. , V. Walter , B. Schottker , M. Jenab , M. G. O'Doherty , F. Kee , et al. 2018 Impact of prediagnostic smoking and smoking cessation on colorectal cancer prognosis: a meta‐analysis of individual patient data from cohorts within the CHANCES consortium. Ann. Oncol. 29:472–483. 29244072 10.1093/annonc/mdx761 PMC6075220

[36] Wang, R. , M. J. Wang , and J. Ping . 2015 Clinicopathological features and survival outcomes of colorectal cancer in young versus elderly: a population‐based cohort study of SEER 9 Registries Data (1988–2011). Medicine 94:e1402. 26334895 10.1097/MD.0000000000001402 PMC4616510

[37] Popat, S. , R. Hubner , and R. S. Houlston . 2005 Systematic review of microsatellite instability and colorectal cancer prognosis. J. Clin. Oncol. 23:609–618. 15659508 10.1200/JCO.2005.01.086

[38] Guastadisegni, C. , M. Colafranceschi , L. Ottini , and E. Dogliotti . 2010 Microsatellite instability as a marker of prognosis and response to therapy: a meta‐analysis of colorectal cancer survival data. Eur. J. Cancer 46:2788–2798. 20627535 10.1016/j.ejca.2010.05.009

[39] Loree, J. M. , A. A. Pereira , M. Lam , A. N. Willauer , K. Raghav , A. Dasari , et al. 2018 Classifying colorectal cancer by tumor location rather than sidedness highlights a continuum in mutation profiles and Consensus Molecular Subtypes. Clin. Cancer Res. 24:1062–1072. 29180604 10.1158/1078-0432.CCR-17-2484 PMC5844818

[40] Sinicrope, F. A. , Q. Shi , C. J. Allegra , T. C. Smyrk , S. N. Thibodeau , R. M. Goldberg , et al. 2017 Association of DNA mismatch repair and mutations in BRAF and KRAS with survival after recurrence in stage III colon cancers: a secondary analysis of 2 randomized clinical trials. JAMA Oncol. 3:472–480. 28006055 10.1001/jamaoncol.2016.5469 PMC5498991

[41] Kerber, R. A. , and M. L. Slattery . 1997 Comparison of self‐reported and database‐linked family history of cancer data in a case‐control study. Am. J. Epidemiol. 146:244–248. 9247008 10.1093/oxfordjournals.aje.a009259

[42] Aitken, J. , C. Bain , M. Ward , V. Siskind , and R. MacLennan . 1995 How accurate is self‐reported family history of colorectal cancer? Am. J. Epidemiol. 141:863–871. 7717363 10.1093/oxfordjournals.aje.a117522

